# Clinical Significance of Nephrogenic Adenoma of the Urinary Bladder: Implications for Differential Diagnosis and Potential for Malignant Transformation

**DOI:** 10.7759/cureus.75094

**Published:** 2024-12-04

**Authors:** Zoran Filipovic, Uros Kojic, Nikola Lukac, Uros Nesic, Djordje Milic

**Affiliations:** 1 Urology, University Hospital Medical Center Bezanijska kosa, Belgrade, SRB

**Keywords:** malignant alteration, metaplasia, nephrogenic adenoma, tumorlike lesion, urinary bladder neoplasm

## Abstract

Nephrogenic adenoma of the urinary bladder is a rare, benign lesion associated with prior inflammation or irritation of the urothelium. Although typically benign, nephrogenic adenoma can present diagnostic challenges due to its potential to mimic malignant tumors of the urinary tract. In this report, we present a case of an elderly woman with a history of recurrent urinary tract infections and bladder stone surgery who developed nephrogenic adenoma. The disease was treated with transurethral resection, which was performed twice during careful follow-up for recurrence. Given the occurrence of recurrence, in both cases during follow-up, the histopathological findings of the removed lesions showed that they were the same type of lesion, nephrogenic bladder adenoma. Given the possibility of recurrence and the rare possibility of malignant transformation, long-term follow-up is essential.

## Introduction

Nephrogenic bladder adenoma is a rare benign metaplastic lesion of the urothelium, also called nephrogenic adenoma, mesonephroid metaplasia, or adenomatous metaplasia [1-3]. Although it is most often observed in the urinary bladder (68.6%), it can also affect other parts of the urinary tract, such as the urethra (13.3%), ureter (8.2%), renal pelvis (8.2%), and in rare cases, the prostate (2%) [2,4]. Research shows that environmental factors and specific lifestyle behaviors, such as cigarette smoking, can contribute to its development [2,3]. This condition is often associated with previous inflammatory processes in the bladder, such as recurrent infections, inflammation caused by bladder stones, foreign bodies, intravesical therapy, chemical exposure, radiotherapy, or previous bladder surgery [5]. Nephrogenic adenoma is of particular importance due to the differential diagnosis, which can favor clear cell adenocarcinoma, endocervicosis, papillary urothelial carcinoma, prostatic adenocarcinoma of the bladder, and nested variant of urothelial carcinoma.

Nephrogenic adenoma manifests clinically with nonspecific symptoms of the urinary tract, with irritative symptoms such as urgency and frequent urination being the most common. Hematuria is uncommon [3,4]. An altered mucosal appearance, which is typically characterized by vesicles and bullae, is typically revealed by cystoscopic examination. This appearance can be closely resembling that of papillary carcinoma of the urinary bladder [4].

Histologically, nephrogenic adenoma is defined by tubular and papillary formations lined by low cuboidal to columnar epithelial cells. Its immunohistochemical profile can vary but is often marked by positivity for paired box (PAX) 2, PAX8, and cytokeratin 7 (CK7), along with negativity for p63 and prostate-specific antigen (PSA) [3,4,6]. 

## Case presentation

**Figure 1:**
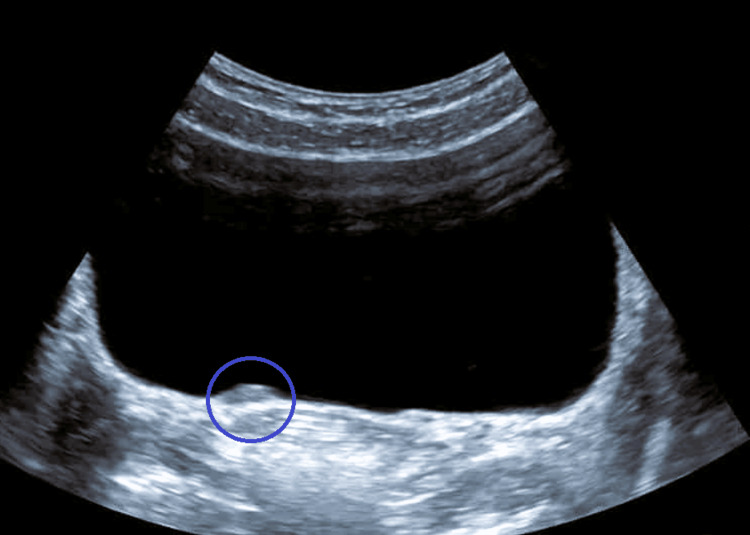
Ultrasound image of the urinary bladder of a patient with NA. Hyperechoic thickening about 10 mm in diameter marked by a circle on the right posterolateral wall of the urinary bladder NA: nephrogenic adenoma

**Figure 2:**
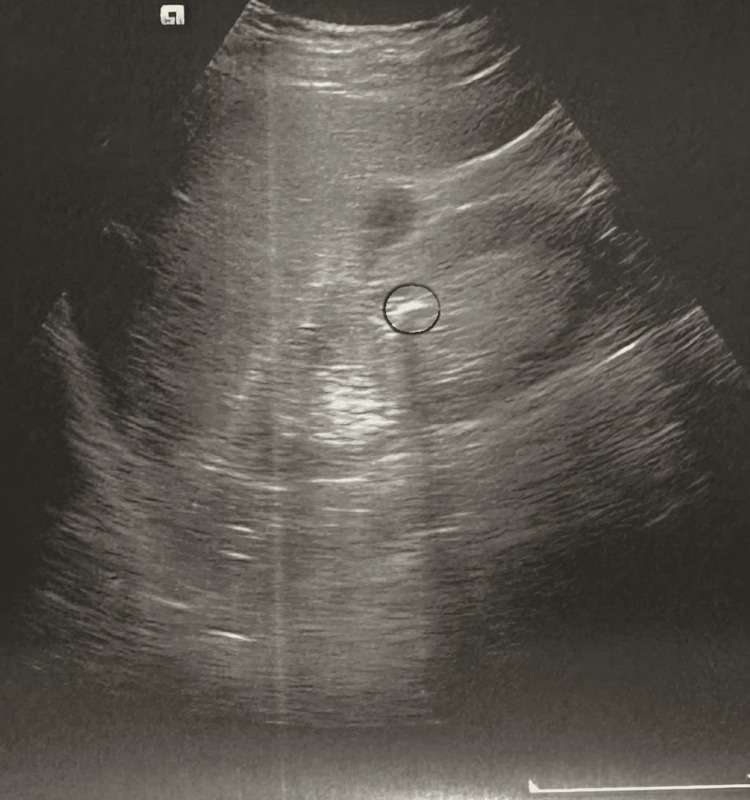
Echosonographic view of the kidneys of a patient who developed a nephrogenic bladder adenoma. The right kidney with an ultrasound image of a stone in the largest diameter of 6 mm shown with a circle

An 84-year-old female patient presented for examination with a history of hematuria and stated that she had been passing sandlike granules in her urine for the past few months. It also provides anamnestic data on recurrent urinary infections. Urinalysis revealed a significant number of leukocytes and 5-10 erythrocytes per high-power field. Echosonographic examination of the urinary tract revealed bilateral microcalculosis up to 6 mm (Figure 1) as well as hyperechoic thickening with a diameter of about 10 mm on the right side wall of the urinary bladder (Figure 2).

**Figure 3:**
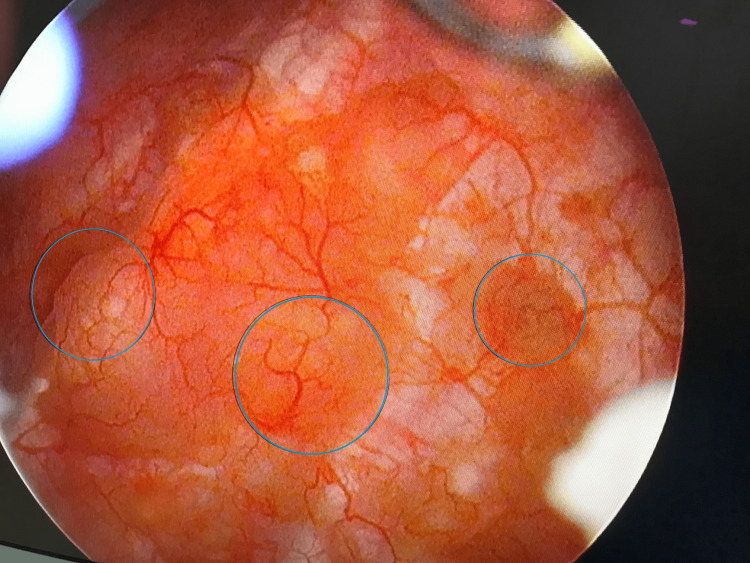
Cystoscopy of the bladder affected by adenoma. Cystoscopic field of view with a circle marked by bullous-cystic hyperemic changes on the right lateral wall of the bladder partially covered by fibrin deposits, indicating a tumorlike lesion

**Figure 4:**
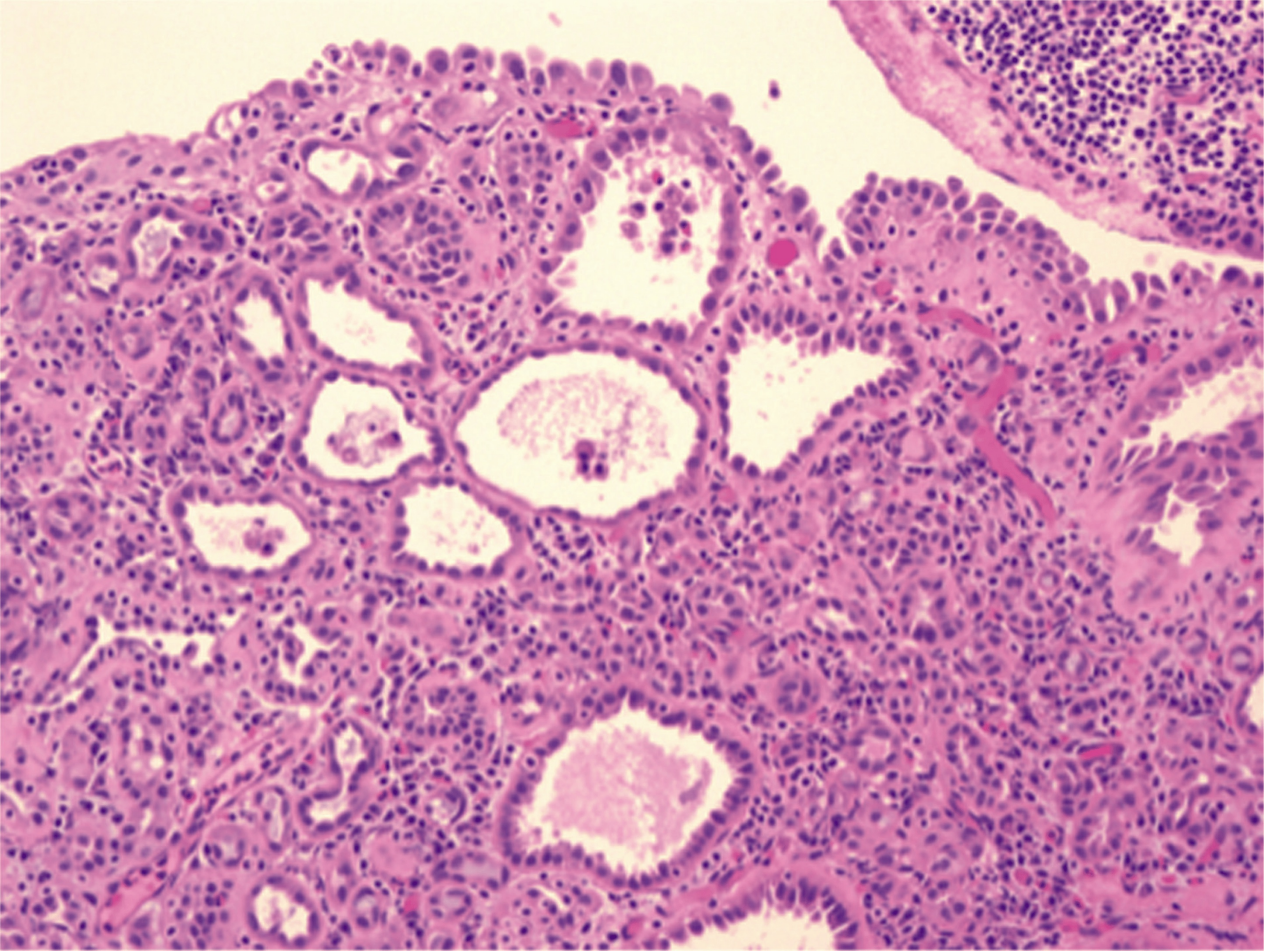
Pathohistological finding of nephrogenic bladder adenoma. Presentation of a chronic inflammatory infiltrate with metaplastic mucosa forming papillary, cystic, and tubular structures, lined by a single layer of cuboidal cells with minimal atypia

Rigid cystoscopy, performed based on prior sonographic findings, showed bullous and hyperemic changes on the right lateral wall, partially covered by fibrin deposits, which were suggestive of a tumorlike lesion (Figure 3). Transurethral resection of the lesion was subsequently performed, along with electrocauterization of the surrounding mucosa. Histopathological examination, including hematoxylin and eosin (H&E) and immunohistochemical staining (AE1/AE3, CK7, CK20, CD34, p63), confirmed the diagnosis of nephrogenic adenoma vesicae urinariae, characterized by chronic inflammatory infiltrates and metaplastic mucosa forming papillary, cystic, and tubular structures lined by a single layer of cuboidal cells with minimal atypia (Figure 4).

At the first follow-up, one month after surgery, the patient reported no symptoms. Urinalysis, which included checking the appearance of color, clarity/turbidity, pH, specific gravity, glucose, ketones, nitrites, leukocyte esterase, bilirubin, urobilinogen, blood, protein, erythrocytes, leukocytes, squamous epithelial cells, crystals, and bacteria, was normal. Microbiological analysis, urine culture, was also performed, which was free of bacterial growth, while urine cytology did not show the presence of abnormal cells. Follow-up with rigid cystoscopy was planned every three months, followed by periodic urinalysis and culture. Although significant bacteriuria was occasionally detected, the patient remained asymptomatic and was treated with non-antibiotic preparations.

Nine months after the first surgery, cystoscopy revealed hyperemic bullous-cystic changes on the posterior wall and roof of the bladder as well as near the scar from the previous surgery. These lesions measured up to 10 mm and bled easily when manipulated. A second transurethral resection was performed, and histopathological findings were consistent with the previous diagnosis of nephrogenic adenoma vesicae urinariae.

The patient has been regularly monitored for 48 months after the second surgery, with rigid cystoscopy performed every six months. No new tumor changes have been observed during this period. Her follow-up regimen also includes urine analysis and culture every three months, as well as periodic sonographic examination of the urinary tract.

## Discussion

Nephrogenic adenoma of the urinary bladder was first described by Davis in 1949 as a bladder hamartoma [7]. Although it is considered a benign metaplastic lesion [1,2,3] due to the possibility of recurrence and the rare possibility of malignant transformation, long-term follow-up is crucial.

This case highlights the importance of accurate histopathological diagnosis and careful follow-up of patients with nephrogenic adenoma due to the lack of standardized treatment protocols. An accurate diagnosis reduces potential diagnostic confusion, as changes that mimic other lesions such as urothelial carcinoma or chronic inflammation can be seen. It reduces the need for additional diagnostic procedures such as imaging and invasive biopsy. It reassures patients and allows them to focus on an appropriate treatment plan, thereby ensuring the preservation of urinary tract function. Misdiagnosis can lead to delays in appropriate treatment and can cause significant psychological distress, leading to anxiety and uncertainty about the prognosis. However, overtreatment of a benign lesion could result in unnecessary surgical risks, prolonged recovery time, and potential complications.

The patient who has been monitored for the last five years shows a recurrence of the disease but without pathohistological findings that would show signs of malignant transformation. This raises the question of whether such transformation occurs at the site of the initial lesion, the nephrogenic adenoma, or whether a new neoplastic formation of the bladder could develop elsewhere.

Vemulakonda et al. [8] reported a case of recurrent nephrogenic adenoma of the bladder in a 10-year-old boy with a history of plum syndrome. They stated in their opinion that their reported case was the first reported case of recurrent nephrogenic adenoma in a patient with a history of plum belly syndrome. While most cases of nephrogenic bladder adenoma have a benign and unrepeatable biological behavior, the conclusion drawn from this report indicates that regular follow-up cystoscopies would be necessary in order not to miss a recurrent nephrogenic bladder adenoma. Boscolo-Berto et al. [9] described a case in which a patient experienced three relapses within the same diverticulum, with an identical pathohistological finding of a nephrogenic adenoma that was treated with transurethral resections. Since they considered the nephrogenic adenoma to be a benign lesion without any direct evidence of possible evolution into malignancy, they conducted several years of cystoscopic follow-up. Their work is also one of the first reports of a nephrogenic adenoma showing recurrence, but the first case of a highly recurrent nephrogenic adenoma in the same diverticulum that was treated conservatively with routine transurethral resection. Hartmann et al. [10] documented the case of a 70-year-old woman who experienced multiple recurrences of nephrogenic metaplasia in the bladder, which eventually led to the development of clear cell adenocarcinoma. The results of the molecular studies they conducted suggested the clonal evolution of nephrogenic metaplasia to the appearance of cellular adenocarcinoma in this case. Hungerhuber et al. [11] reported a 25-year-old man who had trauma to the urinary bladder caused by a traffic accident. After the patient recovered from the accident, he developed a nephrogenic adenoma and recurrent urinary tract infections. Since the accident, he had a nephrogenic bladder adenoma for 18 months. The adenoma was treated several times with transurethral resections. Initial pathological findings were benign; however, the last resection revealed that the previously benign adenoma had transformed into a moderately differentiated bladder adenocarcinoma. Dhaliwal et al. [12] present a rare case of clear cell adenocarcinoma arising in the area of ​​nephrogenic metaplasia. This is an unusual case because the morphology of nephrogenic metaplasia has been seen to change over time with the subsequent development of clear cell adenocarcinoma.

## Conclusions

Nephrogenic adenoma, though benign, presents notable clinical challenges due to its rare but documented risk of malignant transformation and potential for recurrence. Its unpredictable nature combined with the lack of standardized treatment protocols underscores the need for careful, long-term monitoring. While transurethral resection remains the main treatment option, it is not always definitive, as recurrence can still occur even after complete resection. To improve management strategies that address both its benign and possible malignant aspects, further research is needed to better understand the underlying pathogenesis of nephrogenic adenoma. 
